# Supplementary material for: Reconciling Mining with the Conservation of Cave Biodiversity: A Quantitative Baseline to Help Establish Conservation Priorities
Source: PLoS One. 2016 Dec 20;11(12):e0168348. doi: 10.1371/journal.pone.0168348 (PMC5173368; doi:10.1371/journal.pone.0168348)
Supplement: S1 Dataset — (ZIP) [file pone.0168348.s002.zip › Taxa/Serra Norte/SN_2013/N4E/Lista N4E-47.pdf]

CAVIDADE N4E-0047

Classe	Ordem	Fam/Outros	Gên/Outros	Espécie	S-U	U-S	Zon.
Arachnida	Acari	Erythraeidae		sp.64	X		E
Arachnida	Acari	Ixodidae		jovem	X		E
Arachnida	Araneae	Ctenidae		jovem	1		E
Arachnida	Araneae	Filistatidae		jovem		X	E
Arachnida	Araneae	Ochyroceratidae	*Ochyrocera*	sp.2		X	E
Arachnida	Araneae	Ochyroceratidae	*Ochyrocera*	sp.4	X		E
Arachnida	Araneae	Oonopidae	Oonopinae	sp.1	X		E
Arachnida	Araneae	Pholcidae	*Mesabolivar*	sp.1	X	X	E
Arachnida	Araneae	Scytodidae	*Scytodes*	jovem	2		E
Arachnida	Araneae	Theraphosidae	*Gen*.n.	sp.1		1	E
Arachnida	Opiliones	Stygnidae	*Protimesius*	*gracilis*		1	E
Chilopoda	Scutigeromorpha	Pselliodidae		jovem	1		E
Diplopoda	Spirostreptida	Pseudonannolenidae	*Pseudonannolene*	jovem	1		E
Entognatha	Collembola	Entomobryidae		sp.1	X		E
Insecta	Blattodea			jovem	1		E
Insecta	Coleoptera			jovem	X		E
Insecta	Diptera	Chironomidae		sp.		X	E
Insecta	Diptera	Tipulidae	Tipulinae	sp.		X	E
Insecta	Heteroptera	Reduviidae	Reduviinae	sp.	2		E
Insecta	Homoptera	Cixiidae		sp.3	X	X	E
Insecta	Hymenoptera	Braconidae		sp.1		X	E
Insecta	Hymenoptera	Formicidae	*Camponotus*	sp.1	X		E
Insecta	Hymenoptera	Formicidae	*Dolichoderus*	aff. *bispinosus*	X	X	E
Insecta	Hymenoptera	Formicidae	*Hypoponera*	sp.1	X		E
Insecta	Hymenoptera	Formicidae	*Pachycondyla*	sp.1		X	E
Insecta	Hymenoptera	Formicidae	*Paratrechina*	sp.1	X		E
Insecta	Hymenoptera	Formicidae	*Paratrechina*	sp.2	X		E
Insecta	Hymenoptera	Formicidae	*Pheidole*	sp.2		X	E
Insecta	Isoptera	Termitidae	*Embiratermes*	sp.	X	X	E
Insecta	Lepidoptera	Gelechioidea		sp.	X		E
Insecta	Psocoptera			jovem		X	E
Insecta	Thysanura	Nicoletiidae		sp.1	X		E
Gastropoda	Pulmonata	Systrophiidae	*Happia*	sp.		X	E
Malacostraca	Isopoda	Dubioniscidae		sp.1	X	X	E
Malacostraca	Isopoda	Philosciidae		sp.2		X	E
Turbellaria	Tricladida			sp.2		2	E
Mammalia	Chiroptera	Emballonuridae	*Peropteryx*	*kappleri*	1		E
